# Multidisciplinary database of permeability of fault zones and surrounding protolith rocks at world-wide sites

**DOI:** 10.1038/s41597-020-0435-5

**Published:** 2020-03-19

**Authors:** Jacek Scibek

**Affiliations:** Earth and Planetary Sciences, McGill University, Montreal, QC Canada

**Keywords:** Structural geology, Hydrology

## Abstract

Brittle faults and fault zones are important fluid flow conduits through the upper part of Earth’s crust that are involved in many well-known phenomena (e.g. earthquakes, thermal water and gas transport, or water leakage to underground tunnels). The permeability property, or the ability of porous materials to conduct water and gas, is one of the key parameters required in understanding and predicting fluid flow. Although close to a thousand studies have been done, and permeability tested in parts of fault zones, a sytematic summary and database is lacking. This data descriptor is for a multi-disciplinary world-wide compilation and review of bulk and matrix permeability of fault zones: 410 datasets, 521 reviewed sites, 379 locations, >10000 publications searched. The review covers studies of faulting processes, geothermal engineering, radioactive waste repositories, groundwater resources, petroleum reservoirs, and underground engineering projects. The objectives are to stimulate the cross-disciplinary data sharing and communication about fault zone hydrogeology, document the biases and strategies for testing of fault zones, and provide the basic statistics of permeability values for models that require these parameters.

## Abstract (summary)

**Table:** 

Measurement(s)	permeability
Technology Type(s)	digital curation
Factor Type(s)	data source • geographic location
Sample Characteristic - Environment	geological fault

Machine-accessible metadata file describing the reported data: 10.6084/m9.figshare.11933391

## Background & Summary

Geologic evidence of past fluid flow through brittle fault zones is abundant and varied in fault rocks and adjacent altered host rocks^1–3^. The hydrogeological effects on fluid flow in fault zones (e.g. barriers and conduits) have been observed directly at present time. These include thermal springs discharging from faulted crystalline rocks^4^, faults distrupting fluid flow in sedimentary rocks because of formation offset and clayey fault gouge seal^5^, or groundwater inflow to underground excavations from fractured rocks in fault zones^6,7^. Faults or fault zones are now recognized as heterogeneous domains of deformation and associated hydro-mechanical properties from macroscopic to microscopic scale, that may include: single or multi-strand fault core, damage zones of fractured rock, deforming and off-setting the surrounding host rocks or protoliths^8–11^. The brittle deformation is also recognized at microscopic or microfracture scale around faults^12^. Permeability is an important parameter in models of active faults because of fluid involvement in fault geomechanics and earthquake generation^13–16^, and temporally enhanced permeability is now directly linked to some types of observed migrating seismicity phenomena along faults^17,18^. The bulk permeability of the brittle upper crust is only partly known, but there is multiple evidence for fault zones behaving as conductive elements or fluid flow channels through the brittle crust^19^. The paleo-permeability of faults can be inferred from the properties of fault-hosted ore-deposits^20,21^. At present time, hydrothermal flow systems in explored geothermal reservoirs often involve permeable faults and thermal fluid convection within fault zones^22,23^, and both locally and regionally the contrast of fault-host rock bulk permeability is a controlling parameter^24^. Some of the best (or the most specific) permeability data are from fault-hosted geothermal reservoirs^25,26^. In sedimentary basins, fault permeability and transmissivity in petroleum reservoirs is intensely studied because of fluid compartmentalization effects^27,28^, but these effects are also noted in shallow groundwater aquifers^29^. Fault conduits and barriers to water flow also affect the engineering of underground excavations^30^, and predictions of contaminant transport.

The published data from fault zones are often isolated from each other in the sub-specialties within the geoscientific disciplines, and not cross-referenced, and continuously diverging into more specialized fields of study. With a growing number of measurements, data compilation becomes difficult, while the geoscience specialization can lead to certain biases in data collection at different scales of measurement^31^. The multidisciplinary data search is important because most authors in particular geoscientific specialization do not reference works from outside of their field of study, thus creating rather limited reference links that tend to be re-used by subsequent authors.

The compilation and description of these permeability data has several objectives:

1. improve cross-disciplinary data sharing and communication, and serve as a reference or a guide to the multitude of datasets,
2. present summary values and site information for as many as possible published datasets and not only selected sites,
3. enable the statistical exploration of fault zone permeability with structural and geologic parameters for the study sites,
4. compare the magnitudes of permeability in fault zones and host rocks,
5. stimulate a critical discussion of this topic, and offer improvements in dataset review and database expansion.

**Fig. 1:**
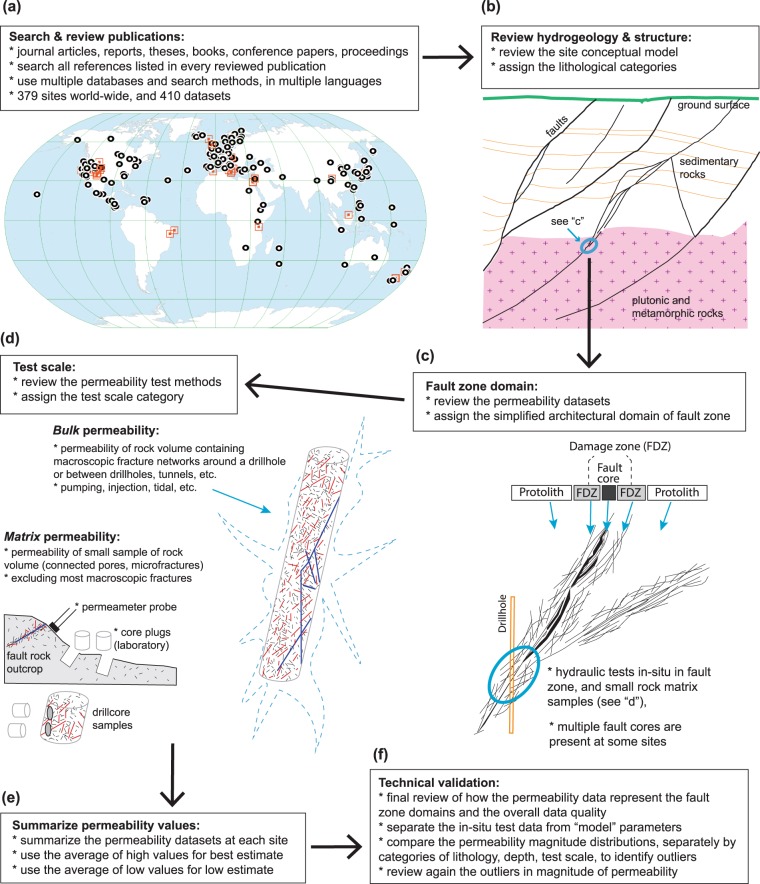
Permeability test data review process diagram. Schematic diagram of the permeability data compilation and review process: (**a**) search and review of publications (map of the world showing the locations of test sites in this study), (**b**) hydrogeology & structure at site scale (sketch based on Soultz-sous-Forêts geothermal site modified after ref. ^116^, (**c**) fault zone structure and permeability domains (fault zone sketch modified after ref. ^117^), (**d**) permeability test scale (drillhole fracture model modified after ref. ^118^), (**e**) summary of permeability values in database, (**f**) technical validation.

The data descriptor in this article^32^ is for a database of world-wide test sites in brittle fault zones (test methods, geologic parameters, summarized permeability values), at different geographic locations (Fig. 1). The review was done over seven years, in several iterations, and covered thousands of research items such as journal papers, reports, and conference proceedings published until February 2020. Of the reviewed datasets, 410 passed the initial review, and 111 datasets did not meet the criteria because of lack of published permeability data or unclear data association with fault zones.

## Methods

### Dataset review process

The concept of “systematic review” and meta-analysis is used in many scientific fields^33^, and here it was adapted to the hydrogeological data that are both qualitative and quantitative. The search was comprehensive, using multiple databases, and then searching and reviewing all references listed in every publication on the topic of fault zone permeability. The full search of reference lists in all publications was ultimately more systematic and complete, compared to the searches through academic journal or institutional report databases, partly because the titles and keywords were moderately to poorly useful in locating the permeability datasets or supporting documents. The review process is summarized in Fig. 1, and described in the following text sections.

### Data sources

The data sources are organized in six categories of “Category of research”, defined loosely by the objectives and motivations of the studies, although many of the studies were multi-disciplinary (Table 1**)**. A list of named fault zones, research sites, geothermal reservoirs, tunnels, mines, outcrops or named drillholes is in Online-only Table 1, to help with document and publication searches for these terms.

**Table 1:** Dataset counts by category of research.

Category of research	Reviewed datasets	Datasets passing review	Downhole *in-situ* tests	Outcrops only
Active faults and faulting processes	120	92	59	33
Geothermal reservoirs	169	115	115	0
Radioactive waste repositories	64	61	58	3
Water resources and contaminated sites	27	21	19	2
Petroleum reservoirs and faulting processes in sedimentary rocks	67	57	14	44
Engineering projects (tunnels, mines, dams)	74	65	65	0
Totals	521	411	331	35

A dataset may include one or many permeability tests and results, and multiple types of methods.

#### Active faults and faulting processes

Approximately 2500 research items were reviewed from structural and hydrogeological studies at 79 locations and 120 datasets. Representative fault zone permeability values were entered for 92 datasets. The database also contains several estimates of paleo-permeability from fault outcrop mineralogy and geometry of sealed fractures, clearly separated from the *in-situ* test results for present-day permeability. A large number of fault outcrops have been mapped, but relatively few locations have been tested for permeability. The data sources are varied and are listed as follows.

Scientific drilling and outcrop testing:

- permeability testing of fault rocks on outcrops of active fault zones in metamorphic and plutonic rocks^34,35^,
- testing of fault outcrops in volcanic rocks^36,37^,
- drilling and *in-situ* hydraulic testing in parts of active fault zones on continents^38–41^,
- tests in tunnels through parts of active faults^42^,
- tests on drillcore samples retrieved from depth^43–45^,
- oceanic drilling program and fault permeability estimates at various locations in the accretionary prisms^46,47^,
- deep scientific drilling in metamorphic crust^48–50^,

Seismological observations and fluid flow models:

- permeability of fault conduits inferred from the rate of migration of seismicity near fluid injection locations^51,52^,
- permeability and poro-elasticity of host rocks with various effects of faults on fluid flow^53,54^,
- natural seismicity migration in faulted brittle crust^55,56^,

#### Geothermal reservoirs

The review was initially done on published data from 240 locations, then refined to 169 datasets. However, after the final review, only 115 permeability datasets were clearly fault-related. Particularly useful sources of geothermal data were: the UNU-GTP (United Nations Geothermal Training Programme) in Iceland, the GRC (Geothermal Resources Council) database, and the Stanford University Geothermal Workshop proceedings. The review and permeability summaries were also done for EGS (Enhanced Geothermal System) and HDR (Hot Dry Rock) sites. The enhanced permeability of faults after pressure injection (“fault stimulation”) were stored separately from the values “before stimulation” (natural conditions). In many geothermal fields, the numerical fluid flow models provide a good summary of site-wide bulk permeability, because the models are calibrated to *in-situ* hydraulic tests. In some cases, original *in-situ* data were not published, only the site-wide models.

Examples are:

- enhanced geothermal reservoirs^57,58^,
- geothermal exploration and production in natural hydrothermal areas, including fault-hosted geothermal reservoirs^59,60^,
- faulted volcanic and sedimentary reservoirs where the fault conduit-barrier effects are complicated by and permeable stratigraphic units^61,62^,
- upflow of thermal waters in natural hot geothermal areas^63^,

#### Radioactive waste repositories

The hydrogeological investigations were motivated by characterization of proposed radioactive waste disposal sites, usually located in low-porosity fractured and faulted metamorphic, plutonic, and volcanic rocks (in total 61 datasets). The reports containing the data are searchable through the INIS Repository database of the International Atomic Energy Agency (IAEA) and from national agencies such as Nagra (Switzerland), SKB (Sweden), Posiva Oy (Finland), JAEA (Japan), AECL (Canada), and the USGS and U.S. Department of Energy, as well as national laboratories. Selected results have also been published previously in scientific journals. The fault structures are often old inactive thrust faults or ductile shear zones or minor faults in the regional setting, because these locations were purposely selected to be in less deformed tectonic blocks. Examples of datasets are:

- metamorphic (e.g. metasedimentary gneisses) and plutonic rocks (e.g. granitic batholiths)^64,65^,
- mudrocks (e.g. argillites, shales)^66,67^,
- volcaniclastic rocks (e.g. tuffs)^68,69^,

#### Groundwater resources and contaminated sites

Aquifer models average large volumes of material, and in a few cases, there are estimates of fault zone conductance (or hydraulic conductivity) for groundwater flow across the fault, calibrated to the observed hydraulic head distributions around the fault zone. More definitive *in-situ* tests were done at some research sites in combination with tracer tests and detailed flow models. Examples of datasets are:

- groundwater resources in fractured and faulted metamorphic or plutonic rocks^70^,
- groundwater flow in faulted siliciclastic sedimentary rocks^71^,
- effects of karst dissolution on fault zones in carbonate rocks^72^,

#### Petroleum reservoirs and faulting processes in sedimentary rocks

Many permeability measurements are done routinely by the petroleum industry, and the search results were from publicly available articles and reports only. The published data are largely from fault zones exposed on outcrops, and less from drilled study sites. Examples are:

- fault outcrops in sandstones^73,74^, and in carbonates^75,76^,
- tests on of drillcore samples^77^,

#### Engineering projects

This section relies upon reports and papers on hydraulic tests and observations in long tunnels, mines, and dams constructed in various rock types at depths up to 2 km. The groundwater inflow to tunnels from fault zones can provide estimates of transmissivity and bulk permeability. Examples of datasets are:

- hydrogeology of fault zones in deep transportation tunnels, tested using drillholes^78^,
- estimates of fault zone and host rock permeability from water inflow rates along tunnels^79^,
- inflows from faults to mines^80^.

**Fig. 2:**
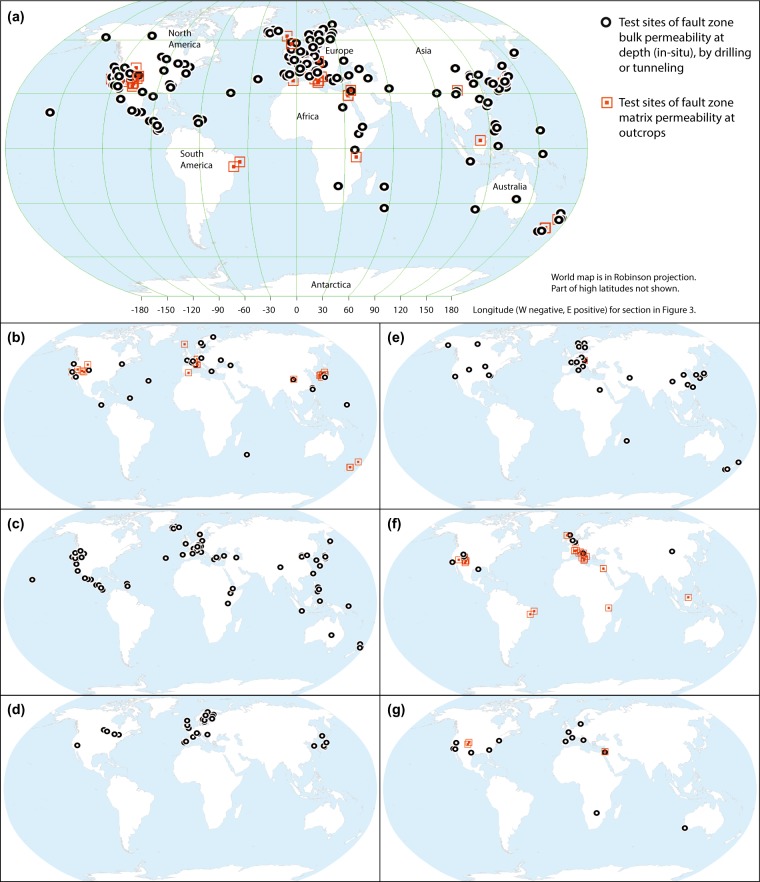
Locations of world-wide fault zone permeability test sites. (**a**) Locations with available bulk and matrix permeability, (**b**) datasets from studies on active faults and faulting processes, (**c**) geothermal reservoirs, (**d**) radioactive waste repository studies, (**e**) groundwater resources and contaminated sites, (**f**) petroleum reservoirs and faults in sedimentary rocks, and (**g**) engineering projects.

The locations of reviewed test sites on world maps are plotted in Fig. 2. The geographic coordinates were either reported in the original publications, or, were estimated from published maps on figures in those publications. For each study site, the location was checked in Google Maps or Google Earth applications, compared to the descriptions in published reports or papers. A note was entered in the database about location uncertainty.

### Geologic parameters

**Fig. 3:**
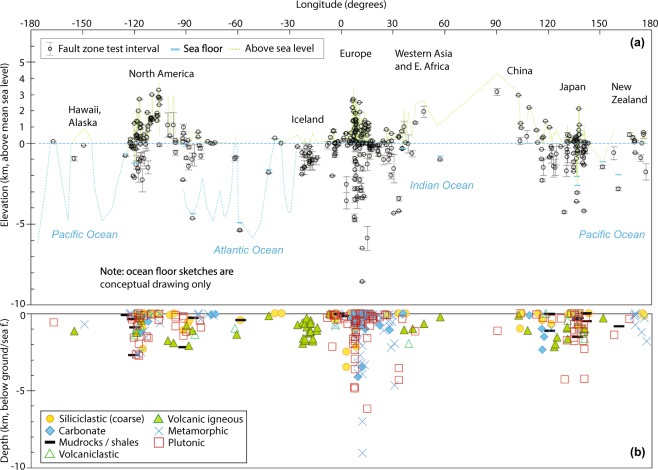
Fault zone permeability test point elevations and depths plotted along geographic longitude. (**a**) Test sites plotted as ground or sea floor elevation, (**b**) depth below ground or below sea floor of *in-situ* bulk permeability test intervals by host rock lithology category.

As a broad overview, all test sites are in the upper part of the brittle Earth’s crust, in both the crystalline “basement” rocks and in the overlying sedimentary basins or volcanic rocks. The depth intervals of tests in fault zones can be visualized along a schematic cross-section along the geographic longitude (Fig. 3), where all the data are projected on the section and shown as elevation above sea level or as depth below ground or sea floor. The depth range of *in-situ* tests in drillholes or tunnels is from 0 to about 9 km depth below ground on land, and to about 1 km depth below sea floor at ocean drilling sites.

A brief summary text of the local geology and regional context is included in the database. The host rocks were classified into categories by their overall rock type and not particular mineralogical characteristics:

- “Siliciclastic (coarse)” category (n = 89) includes the sandstone and interlayered sandstone with fine grained rocks. A sub-category was given for unlithified siliciclastic rocks, and a category for tests in deformation bands (reviewed in detail by other authors^81^).
- “Mudrock” category (n = 30) here refers to the fine-grained siliciclastic rocks^82,83^ clay, silt, claystone, mudstone, clay-rich siltstone, shale, and any argilliceous rocks). Marl rock and mixed mud-carbonate rocks, that have relatively low strength and smear when faulted, were placed in the mudrock category due to their hydro-geomechanical properties^84^.
- “Carbonates & evaporites” category (n = 47) is for the test sites mainly in limestone or dolomite, often metamorphosed to some degree. At a few locations the dolostones are interbedded with anhydrites and other evaporites or marble. A few outcrops were in high-porosity grainstones.
- “Volcaniclastic” category (n = 25) has the test data mainly from tuff rocks and tuffaceous sediments. There is a large variability in hydraulic properties of tuffs, depending on their cementation and alteration^85^. In volcanic areas, tuffaceous rocks are usually interlayered with andesitic or basaltic layers, thus in some cases both categories of volcaniclastic and volcanic igneous were marked as present.
- “Volcanic igneous” category (n = 73) is for the intrusive and extrusive volcanic rocks, although these can be interlayered with volcaniclastics and other sediments.
- “Plutonic” category (n = 137) here is for the granitic and granodioritic rocks from a wide variety of data sources: drilling in large batholiths (e.g. exposed in mountain massifs) or at smaller intrusions at some geothermal fields.
- “Metamorphic” category (n = 126) is a large group of metamorphic rocks that are found in the basement rock at most of the study sites.

It should be noted that host rocks around faults also contain an agglomeration of deformations that may not be related to the presently tested fault zone (e.g. sparse natural fractures of various origin, intrusive dykes, stratigraphic or lithologic contacts and associated fracturing, old ductile deformation that is now part of the host rock for the brittle fault, etc.). The host rocks that are considered here are with respect to the now (in most cases exhumed) brittle fault zones. This review does not cover ductile shear zone permeability or their protoliths at the time of shear zone activity.

The structural geology was reviewed for each site and the available fault size parameters were summarized: fault displacement and throw, length, width of fault core and damage zone, and whole fault zone width. The total displacement was rarely reported, and it was found at about 25% of the sites. The total displacement for a fault zone is often accumulated among many fault segments within fault zones. Fault throw, or dip-slip displacement, was entered separately. Fault length was either for the whole fault zone or for fault segment in the fault zone (if tested specifically). The fault length, where not reported in publications, was estimated in this review from the geologic maps and figures in some cases. The best mapped widths of fault zone components were the fault cores. Fault core width varied greatly from cm to multi-meter scale at many study sites, and many of the fault zones had multiple fault cores (multiple fault strands). The closest fault core to the one tested for permeability was reported here. In most cases, there was no specific fault core tested, and thus the widest reported fault core width was entered to this database. The width of fault damage zone is difficult to define at the majority of study sites. Fault zone widths are only certainly known across exposed fault outcrops or along tunnel section. The width value can be viewed as a sample from a width population along the fault.

### Review of permeability test methods

In each case study, the permeability estimates were associated with particular test methods in the database. This is coded by categorical variables (1 = yes, blank = no) and text summaries. The first group of methods involves some direct estimation of permeability through Darcy’s Law, by inducing a pressure gradient and fluid flow through porous media. The second group is for other estimation methods that require more assumptions about how the observed response variables relate to permeability of porous media. Most of the methods apply to present-day permeability, with exception of the geochemical/mineralogical estimation of paleo-fluid flux (column 64). The method of fracture aperture empirical laws (column 61) was also in some cases applied to mineral-filled (sealed) fractures to estimate paleo-permeability that is obviously different from present permeability of such rock.

The scale of permeability tests is important to differentiate, because the rock matrix permeability can be 2 to 4 orders of magnitude smaller than the bulk permeability of the same rock that includes the macroscopic fracture networks in low-porosity brittle rocks^86,87^. In high-porosity weak rocks the fractures add some permeability component^88^, but the fluid-induced transient fracture dilation effects are also important. The relative effect of macroscopic fractures become more important in rocks of lower porosity and larger density^89^. In this compilation, the *matrix* permeability refers to tests on small (few cm size) rock samples or rock outcrop spots, but does not include macroscopic fractures at scales >0.1 meter. The *bulk* permeability refers to an estimate of total permeability from *in-situ* tests (e.g. hydraulic tests in drillole intervals) at length scales of meters to 100’s meters usually. The bulk permeability includes the effects of connected flow channels within macroscopic fracture networks. The terminology is widely used in hydrogeology and structural geology^90–92^.

The matrix permeability is determined directly from tests that induce some water or gas flow across the samples (laboratory permeameter apparatus), or into spots on samples or rock outcrops (permeameter probes). Macroscopic fracture channels or macropores were sometimes tested on whole drillcore samples. The following text is a list of test methods, and include the number of datasets in brackets (not the individual tests or samples). Additional references are given with examples from fault zones, if not already listed in the methods section.

The direct permeametry estimation methods are:

- Core plugs or smaller samples tested inside laboratory permeameters under confining pressure in order to seal the sample, or purposely at high confining pressure to simulate a larger effective depth than the original sample depth (n = 103 datasets),
- Whole drill-core pieces with faults tested inside permeameters^93^ (n = 36),
- Outcrop transects using gas injection or air extraction at test spots (transient or steady state flow rate), including spots on drillcore pieces^94^ (n = 31),

Indirect matrix permeability estimation methods are:

- Microfracture or pore geometry^95^ relations that link the porosity to permeability, or the microfracture size to permeability (n = 8),
- Geochemical/mineralogical methods for estimating the required paleo-permeability^96^ (matrix or bulk, depending on context) of the main fluid flow conduits from larger depths (n = 2),

Bulk permeability direct testing methods, in column order from the database are:

- Infiltration or exfiltration at the outcrop^97^, where water is injected or infiltrated or removed from the outcrop (n = 4),
- Water inflow from the fault zones to tunnels^30,79^ (n = 43),
- Single borehole tests are the most extensively used methods in fault zones and host rocks (injection, discharge, slug or pulse tests)^98–100^ (n = 206),
- Tidal analysis of the water pressure fluctuations in drillholes as a response to the solid earth tides or ocean tides^101^ (n = 10),
- Barometric fluctuations in the unsaturated zone to estimate air flow rate along the fault zones^102^ (n = 1),
- Cross-borehole tests in fractured zones, where the fracture networks are tested directly between two drillholes^38,103^ (n = 39),
- Pumping or injection tests and monitoring of pressure responses in other drillholes (interference tests)^70,104,105^ (n = 139),

Numerical flow models are used at almost all research and industrial sites, and in this review the bulk permeabilities of fault zones and host rocks were useful because the original data (individual well test results) were often not published. Measurements that help calibrate numerical flow models were described for some test sites:

- Water inflows/outflows in the drillholes (circulation loss or well feed points), are used as a supporting information in interpretations of well tests (n = 39),
- Reservoir production/reinjection data (pressure, fluid flow rate, and temperature), used for the numerical flow model calibration (n = 28),
- Tracer tests to estimate the porosity and connectivity of the groundwater flow system^97,105^ (n = 27),
- Spring water or gas flow from faults as a supporting evidence for fluid flow in fault zones^106^ (n = 13),

Other indirect permeability estimation methods:

- Empirical laws for fracture aperture and spacing, used to estimate the bulk permeability^107^ (n = 7),
- Grout injection volume patterns across a fault zone^108^ (n = 3),
- Geophysical methods in drillholes to estimate the permeability (e.g. tube wave reflections)^109^ (n = 3),
- Microseismicity hypocenter migration over time, where the bulk permeability of presumed fluid flow conduits along fault zones is calculated from the estimate of hydraulic diffusivity^110,111^ (n = 15),
- Thermal water upflow velocity that is estimated from the vertical temperature gradient and an estimate of permeability from thermal water outflow rate^112^ (n = 2).

### Depths of tests

#### *In-situ* tests of bulk permeability

All *in-situ* bulk permeability values are assigned a vertical depth below the ground surface on land (or sea floor for oceanic drill sites). For depth intervals, both the maximum depth and minimum depths are specified (columns 75 to 76). For single depth points (not an interval), only the maximum depth is given. The average depth (column 77) here means the mid-point depth between the maximum and minimum depth. It equals the maximum depth for single depth points. In geothermal reservoir models, tunnels, or large research facilities with many drillholes, the depth was given as a range of results from the fault zone (as described in published materials). In mountainous terrain, the average depth below ground was estimated from geologic cross-sections and the published test depths.

#### Matrix permeability from outcrops and samples

For fault zone outcrops on the ground surface, for data storage and graphing purposes a 1 m depth is entered as the maximum depth. This allows to plot the permeabilities of outcrops and *in-situ* data from depths on logarithmic depth scales (instead of 0 m depth of outcrop). For drillcore samples, the depth of each sample was entered as the maximum depth only.

#### Effective depth of matrix permeability for rock samples

A depth value must be associated with the permeability results, but in  many laboratory tests on rock samples a high confining pressure (and effective pressure) is often used that acts on the porous media and compresses it. The matrix permeability tends to descrease with increasing confining pressure (pore space and microfracture compaction), and does not recover completely during depressurization^113,114^. For this review, the matrix permeability values are taken at the smallest reported confining pressures (columns 82 and 83 give the range) in the initial pressurizing path before sample compaction, or as recommended by the authors of publications. The “effective” depth value (column 88) is not the depth of original rock sample (drillcore or outcrop). Effective depth depends on the assumed average pressure gradients in the Earth’s brittle crust, assuming hydrostatic conditions and average pressure gradients^115^ in Eq. (1):

1 $${D}_{eff}=\frac{{P}_{eff}}{(\Delta {P}_{lith}-\Delta {P}_{hydro})}$$

where D_eff_ is the effective depth of permeability sample (km), P_eff_ is the effective confining pressure (MPa) on test sample (see column 87), ΔP_lith_ is the lithostatic pressure gradient (MPa/km), ΔP_hydro_ is the hydrostatic pressure gradient (MPa/km). The assumed average lithostatic pressure gradient was 23 MPa/km and a hydrostatic gradient of 10 MPa/km, thus an effective pressure gradient 13 MPa/km. If the sample drilled depth (or outcrop 0 depth) is much greater (>1 km) than the effective depth, the value in column 89 is tagged as 1. These values can be adjusted by the database users if the site-specific pressure gradients are known more precisely.

### Permeability values

The permeability data were separated by test scale category (*matrix* and *bulk*). The test results that were originally reported in the literature as transmissivity, hydraulic conductivity, or permeability thickness, were converted to m^2^ permeability units. Logarithms of permeability (log_10_ m^2^), were also calculated to aid in graphing of permeability vs. depth, and for further statistical analysis.

#### Representative permeability estimate

Only the summary (representative) values of permeability for datasets are reported in this database, not all individual test points in large datasets from the original publications. This database does not compile all raw data and all test spots on outcrops, multiple drillhole test intervals, etc. For each dataset, the representative permeability values are recorded. At 81% of the study sites there is only one permeability test result from *in-situ* test, and usually only one estimate of bulk permeability in the fault zone. Multiple datasets of fault zone permeability were defined at 19% of the sites, separated for different reasons: distinctly different fault zones, different host rock along the same fault zone, large depth separation of groups of *in-situ* tests, or statistical and model summaries of different fault sets as defined by the authors of original publications.

- The representative permeability was in the majority of cases the only value available.
- Where a larger amount of drilling has been done, the review was focussed on finding the representative bulk permeability values that had been already reported by the original authors. This review uses the published conceptual models and statistics and does not re-analyse the data.
- Where only a few test values were taken, the more recent or higher-quality results were used. Given a choice of two permeability estimates, or two clusters of permeability values in original publications, the maximum value was chosen as representative, and the lower estimate was entered separately in the database.
- The summaries of matrix permeability were provided, not individual test measurements (or other raw data).

#### Low estimate of permeability

Since many drillholes tend to miss the most permeable conduits in fault zones, and some fault rocks from fault core are relatively more lithified or re-cemented, another summary value is the “low estimate” of permeability. In cases of more than one data value (e.g. many drillholes or points along outcrop transect), the low estimate of matrix permeability is as reported on published graphs or tables by other authors, or the average for a cluster of values in the low range of permeability for that site. For example, along an outcrop, a typical low permeability value in the damage zone, but higher than the host rock “background” value, was summarized.

#### Assignment of permeability to fault zone structural domain

The matrix permeability on samples or outcrop spots was assigned either to the fault core, the fault damage zone, or the host rock, as described in the published sources. It must be noted that in drillcore or core plug samples, the damage zone contains macroscopic fractures that are nearly all sealed by the cementing minerals, and the microfractures may be also partially open. Aside from the obvious sampling and scale effects of these tests, the locations within the fault zone were usually clear. The values were entered to columns 90–91 (fault core), 94–95 (damage zone), 100–101 (host rock).

The bulk permeability from *in-situ* tests (drillholes, tunnels, etc.) is the sum of contributing flow paths, dominated by macroscopic flow channels or highly porous rocks, in some rock volume that is influenced by fluid flow of the *in-situ* test. The permeability values were assigned as follows:

- host rock: *in-situ* tests away from the fault core or fault damage zone (values in columns 102–103),
- fault core zone: clear evidence that the fault core was isolated *in-situ*, or that it contributed most of the fluid flow (values in columns 92–93),
- fault zone* refers to some part of fault zone in general (except fault core where isolated in the test interval): values entered to columns 96–97, and then explained by two categorical variables (columns 98 and 99),
- if the tested rock volume is only within the fault damage zone, column 98 has a value 1 and column 99 has a blank value
- if the tested rock volume is within parts of the fault damage zone and may include the main fault core zone, whether or not the fault core contributes significantly (positively or negatively) to the bulk permeability, column 99 has a value 1 and column 98 has a blank value

#### Permeability ratios

Ratios were calculated between bulk permeabilities of the fault zone*, the fault core, and the host rock (protolith), as in Eq. (2):

2 $$permeability\,ratio=lo{g}_{10}\left(\frac{{k}_{1}}{{k}_{2}}\right)$$

where k_1_ and k_2_ are permeability values in m^2^ units. For example, k_2_ is usually the host rock permeability and k_1_ is the fault zone permeability. This ratio can be used to quantify the “conduit” magnitude or “barrier” magnitude of a fault zone, but both the fault zone (or a component of fault zone) and the host rock permeability must be known at the same location. Host rock from distant locations or regional averages cannot be used because the range of permeability values is too wide regionally. In other words, the data must be matched to the same fault zone or part of fault zone at the same location, ideally along the same drillhole or outcrop transect, but adjacent drillholes may be used. The measurement scale must also be consistent (e.g. cannot use matrix permeability for host rock and bulk permeability for fault zone). Both values must be either matrix permeability or bulk permeability, and ideally tested with similar methods and affecting similar rock volumes.

The following ratios were estimated for permeabilities for the best representative value and the low estimate:

- log(bulk k Fault Zone/bulk k Host Rock) (columns 104–105)
- log(matrix k Fault Core/matrix k Host Rock) (columns 106–107)
- log(bulk Fault Core/bulk Host Rock) (columns 108–109)

## Data records

The data repository items^32^ at figshare contain a database with 121 different data fields (or columns), as described in Online-only Table 2. In the spreadsheet version (Microsoft Excel.xlsx file), the first row contains the labels or headings for the columns. The.pdf files contain the printed-out versions of the database and the list of selected references. The following additional notes refer to the spreadsheet table.

Columns 1 to 13 contain information about the study sites and their locations. The first column has the “Dataset number”. Fault zone sites at different geographic localities differ by whole integer numbers. Where there is more than one dataset at a fault zone test site, the dataset number is incremented by the first decimal digit. For example, one site with three datasets on fault zone permeability may have dataset numbers 1.1, 1.2, 1.3, and next site with one dataset is 2. The order of Dataset numbers is by “Category of research” (column 2), next alphabetically by “Country” (column 4), and lastly by “Site name” (column 3). The geographic coordinates are latitude and longitude for the test site (not individual test intervals). Ground surface elevations also apply to whole study sites (e.g. elevation at well-head, topographic elevation above geothermal reservoir or tunnel, sea floor surface at oceanic drillhole, etc.). Sub-sea drilling locations have elevations below sea level (negative values). The elevations are rounded off to match the value uncertainty.

The database columns 14 to 24 describe the host rock type and local geology, as brief text descriptions and categorical variables (1 = yes or present, and blank otherwise) for host rock types. The fault types and sizes are summarized, where known, in columns 25 to 43. There is a referenced text summary about known fault structures. The fault types are categorized (n = counts of test sites): normal faults (n = 180), reverse faults (n = 32), normal-oblique faults (n = 32), strike-sip faults (n = 30), faults that run along dykes or wide veins (n = 30). For some cases, the fault type or size could not be verified at this time. Some faults are simply categorized as high or low angle fault, or a dip value is given (or dip value range). The fault size parameters are in columns 37 to 43. These include any known values of the  fault throw, displacement, length, width of fault core, width of damage zone, or width of whole fault zone.

Permeability test methods are summarized and categorized in columns 44 to 67. A summary text (column 44) is referenced to publications, and with notes about permeability unit conversions. The available permeability values are described in text in column 68, with references to data sources and methods, and notes about any measurement unit conversions. The next column 69 has the result of “Initial review pass” (1 = yes). For selected sites there are comments in column 70 about the review result. Datasets from tests on rock outcrops are tagged in column 72, and those outcrops that have data from fault core or fault damage zone are tagged in column 73. *In-situ* test results from some depth that represent fault zones are indicated in column 74.

## Technical validation

### Qualitative assessment of test site and results

During this review of the published datasets, the conceptual models of fault’s hydrology were compared with the consistency of the results of hydraulic tests in fault zones, and parameters used in calibrated numerical flow models. This qualitative review focussed on several aspects of published data:

- clarity and detail of site descriptions and data analysis methods,
- data quantity (e.g. number of drillholes and tests),
- test methods and scale of tests (matrix vs. bulk permeability),
- site-wide conceptual models, including calibrated numerical groundwater flow models,
- detail of structural mapping and analysis,
- permeability test location within the fault zone or host rocks.

The records that did not pass the review are still kept in the database, in a separate table, because of other useful information about the site. The dataset numbers continue between the two tables, and when merged, are consistent and sequential for the whole database. Perhaps the next review of world-wide permeability test sites may clarify the questions or provide more data.

### Uncertainty of fault size parameters

The fault size estimates are uncertain due to the measurement limitations (e.g. covered outcrops, variable scale of measurement from macroscopic to microscopic). It is often not known what exact fault segments or strands, or overlapping fault damage zones, in a larger fault zone contribute to the bulk permeability in each *in-situ* test. During this review, a statistical exploration was performed of the structural data to compare the fault displacement, length, fault core and fault zone width, and fault type. The apparent outliers were reviewed again.

### Uncertainty of permeability values

The uncertainty of matrix permeability values depends on the test method, and in particular the ability to seal the probe to outcrop or rock sample, the type of fluid used, or sample conditions (damage). Measurement accuracy in laboratory permeameters is usually adequate, but tests on natural fault outcrops are usually not verified for probe seal or gas leakage. Reported values are typically in a range of a factor of 2 to 5 for particular test site.

The uncertainty of bulk permeability values can be attributed to methods of *in-situ* testing, assumptions used in the interpretation of data, and the problems with sampling of highly heterogeneous rocks and fracture networks. For individual well tests, the parameters may be fitted to give bulk permeability within single decimal value, but the results for whole sites are usually considered to be within an order of magnitude of actual conditions. The rock volume that contains a fault zone is many orders of magnitude larger than the volume tested for permeability, thus it is expected that the most permeable or conductive parts may be missed. It is difficult to quantify the uncertainty in bulk permeability of a fault zone. The difference between the representative estimate and the low estimate in this review gives some indication about this uncertainty.

The heterogeneity of hydraulic properties in fault zones cause practical difficulties of separating parts of fault zones for *in-situ* tests, and interpretation of well-test data. The specific *in-situ* data on fault cores (fault core zones) is particularly limited, thus the contributions of fault cores to the total bulk permeability in fault zones cannot be estimated at many test sites.

## Online-only Tables

**Online-only Table 1:** List of fault zone names and names of test sites. The list is organized in sections by category of research.

Fault zone names	Names of test sites
**Active faults and faulting processes**	
Aigion, Alpine, Atera, Atotsugawa, Bersezio, Valetta, Biauhu and Pingshi, Carboneras, Champlain Thrust, Chelungpu, East Fork Thrust, East of Atlantis II, Elkhorn, Gole Larghe, Hanaori (Hanaore), Hayward, Iida-Matsukawa, Jorgillo, Kobus-Lion-Kronthal, Lansjärv, Median Tectonic Line, Minami-awa, Neodani, Neodani, Nishiyama and Tsugawa, Nobeoka Thrust, Nojima-Ogura, Pasmajärvi, Pirgaki, Roccasseira, Roccastrada, Romeleåsen, San Andreas, SEMP, Silver Creek, Stillwater, Stuoragurra, Talhof, Taranaki, Tottori-ken Seibu, Umbria-Marche, Usukidani, Wildcat, Yingxiu-Beichuan (Longmenshan)	Research sites: Coaraze, DGLAB, LSBB, ITA Observatory, Pinyon (Piñon) Flat Observatory, Site de Ploemeur Drillholes or outcrops: IODP U1309D, Auriat, Big Pines, Cajon Pass, Cienega Valley, COSC, Dry Lake Valley, Sage Flat Hollow, Gaunt Creek, Gravberg-1, Hi Vista, Kinbara, Kitagawa, Ankoh, KTB, Logan Quarry, Logatchev 1, Lund, Midori, Mikigadawa, Miyagawa, Mugi, ODP 671, ODP 857D, ODP 892A/892B, ODP 949C/948D, ODP 1117, ODP 735B, ODP 808, ODP C0002, ODP C0009, ODP 1039/1043/1040, ODP C0010/C0004/C0006, Okitsu, SAFOD, Sancerre-Couy, SG-3 Kola, SG-8 Krivoy Rog, Tochudani, Tsukide, Miyamae, Tyrnauz, Waikukupa, Whataroa River
**Geothermal reservoirs**	
Aigion, Boise Front, Chingshui, Hveragil, Leirbotnar, Lorusa and Cougne, Malpais, Namsong, Omerbeyli, Opal Mound, Puhagan, Regua-Verin, Rhyolite Ridge, Slitt Vein, St. Gallen, Stillwater, Takanoyu, Têt, Vallès‐Penedès, Valletta, Wendel, Wonji	Ahuachapan, Akinomiya, Aluto-Langano, Bacon-Manito, Bad Teinach, Balcova-Narlidere, Beowawe, Berlin, Boise, Borax Lake, Botn (Hrafnagil), Bouillante, Brady’s (Bradys) Hot Springs, Bruchsal, Cerritos Colorados, Cerro Prieto, Chingshui, Coaraze, Coso, Crystal Hot Springs, Desert Peak, Dixie Valley, Dubti-Tendaho, Eastgate, Efri-Reykir, Ellidaar, Eskifjörður, Falkenberg, Fenton Hill HDR, Fjällbacka, Gata (Laugaland), Geretsried, Germencik, Glass Mountain, Glerardalur, Groß Schönebeck, Habanero, Hamar, Hatchobaru, Otake, Heber, Hellisheidi, Hijiori HDR, Hjalteyri, Hofsstadir, Kakkonda, Kaldárholt, Kamojang, Kawerau, Kilauea Volcano (Puna), Kizildere, Klamath Falls, Krafla, Lahendong, Landau, Larderello, Las Tres Virgenes, Laugaland (Eyjafjördur), Laugerengi (Ólafsfjördur), Le Mayet de Montagne, Lihir (Ladolam), Lishuiqiao, Long Valley Caldera, Los Azufres, Los Humeros, Makushin Volcano, Matsukawa, Milos, Miravalles, Momotombo, Mori, Mt. Sabalan, Mutnovsky (Dachny), Namafjall (Bjarnarflag), Neal Hot Springs, Nesjavellir, Ngawha, Niutuozhen, Ogachi HDR, Ogiri (Kirishima), Oguni, Ölfus-Bakki, Olkaria East (Olkaria 1), Onikobe Caldera (Katayama), Palinpinon, Pauzhetka (Pautzetsky), Piancastagnaio (Mt. Amiata), Platanares, Pohang, Qualibou caldera, Raft River, Reykjanes, Ribeira Grande, Riehen, Rittershoffen, Roosevelt Hot Springs, Rosemanowes Quarry, Rotokawa, Salavatli, San Pedro do Sul, Selfoss, Silapu, Sorgun, Soultz-sous-Forêts, Steamboat Hills, Sumikawa-Onuma, Susanville, Svartsengi, Terme di Valdieri, The Geysers, Theistarekir, Thelamörk, Tianjin, Tongonan, Uenotai, Unterhaching, Urridavatn, Waldshut-Tiengen, Wendel Hot Springs, Yamagawa, Yanaizu Nishiyama (Okuaizu), Yang Bajain (Yangbajing), Zhangzhou-Fuzhou, Zunil, IODP U1309D, Urach-3
**Radioactive waste repository test sites**	
Bow Ridge, Chalk Cove, Crucifix, Fan Ridge, Ghost Dance, Mozumi-Sukenobu, Ohmagari, Paintbrush Canyon, Tsukiyoshi	Altnabreac Station, Äspö, Atikokan RA, Bátaapáti (Üveghuta), Chalk River RA, Dounreay, Down Ampney, East Bull Lake RA, Finnsjon, Fjällveden, Forsmark, Gideå, Grimsel, Hästholmen (Loviisa), Horonobe, Kamaishi Mine, Kamioka Mine, Laxemar, Mizunami, Mizunami-Shobasama, Mont Terri, Mt. Geumjeong (KAERI), Olkiluoto (ONKALO), Romuvaara, Sellafield, Stripa Mine, Tono Mine, Tournemire, Tournemire, Whiteshell RA, Yucca Mountain, Boettstein, Kaisten, Leuggern, Schafisheim, Siblingen, Wallenberg, Weiach
**Water resources and contaminated sites**	
Banning, Carlsberg, Gloucester, Kojonup, Mission Creek, North Armorican Shear Zone, Pax Mountain, Ramapo, Redstone, Sandwich, Sangre de Cristo, Yair	Byron Superfund Site, Carleton University, Dukwi, Handcart Gulch, Hickory Sandstone Aquifer, La Salvetat Rieumajou, Leadville mine, Leppävirta, Päijänne tunnel, Project Shoal Nuclear Test Site, Santa Susana Field Lab., Savannah River Nuclear Power Plant, Turkey Creek, Cadalso de los Vidrios, Desert Hot Springs, Glenwood Springs, Longano, Oracle, San Luis Valley, St-Brice en Cogles
**Petroleum reservoirs and faulting processes in sedimentary rocks**	
Big Hole, Casaperta, Castle Cove, Cévennes, Clashach, Geleen, Hite, Humur-B, Ispica, Little Grand Wash, Lossiemouth, Maghlaq, Moab, Muweilih, Newport-Inglewood, Nîmes, Olevano–Antrodoco Thrust, Owl Creek Thrust, Peel Boundary, Point Vert, Red, Restefond, Salt Wash, San Ysidro, Sand Hill, Santa Ana, Santa Barbara Channel, Scicli, Thal, Trixie, Victoria Lines	Abiaí, Boncavaï quarry, Bosque, Brejinho, Buckskin Gulch, Canyon Trail, Clashach Cove, Clashach Quarry, Cummingstown, Coquerinho, Elmendorf, Icapui, Le Cros quarry, Leroy Gas Storage Facility, Madonna della Mazza quarry, Margerethen quarry, Miri, Pontrelli and Cantore quarry, Quartier de l’Etang quarry, Ras il-Bajjada, Ras ir Raheb, Tambaba
**Engineering projects (tunnels, mines, dams)**	
Arima-Takatsuki Tectonic Line, Border Ranges, Dewey’s, Frøyfjorden, Goetz, Gryphon, Hermann, Illinois, La Recette, Oslofjord, Otsuka, Periadriatic Line, Puzizhier, Rio Bianco, Rokko, San Jacinto, Scontrone, Wallersbach graben, West Bay, West Rogers	Aica-Mules Tunnel, Aica-Mules Tunnel, Bad Creek Reservoir, Bedretto Tunnel, Brixlegg E. Tunnel, Cleuson-Dixence Gallery, Clyde Reservoir, Con Mine, Diavik Mine, Edgar Mine (CSM), Eklutna Tunnel, El Berrocal Mine, Enasan Tunnel, Fanay-Augeres Mine, Fiskarfjärden Tunnel, Frøya (Froya) Tunnel, Furka Base Tunnel, Giant Mine, Gotthard Base Highway Tunnel, Gran Sasso Tunnel, Hsuehshan Tunnel (Ping Lin), Huilong Hydro Storage Station, Hvaler Tunnel, Kanden-Omachi Tunnel (Kurobe Dam), Klasgarten rockslide, KW Oberhasli Gadmenwasser Supply Gallery, Lindau Dam, Los Ratones Mine, Lotschberg Base Tunnel, Malgovert Tunnel (Tignes Dam), Manapouri Tunnel, Medau Zirimilis Dam, Moawhango-Rangipo Tunnel, Modane Tunnel (Mont d’Ambin), Mont Blanc Tunnel, Monticello Reservoir, Namtall Tunnel, Nibashan Tunnel, Oslofjord Tunnel, Poehla-Tellerhaeuser Ore Field, Rokko Tunnel, Rokko-Tsurukabuto Observation Station (in Rokko rail tunnel), Romeriksporten Tunnel, Salazie-Amont Tunnel, San Jacinto Aqueduct Tunnel, Schwartzwalder Mine, Semmering Base Tunnel, Seyahoo Dam, Sta. Maria-Nalps Water Gallery, Straight Creek Tunnel, Taining Tunnel, Tomitaka Mine, Triadelphia Reservoir (CAES/UPH), Unteralpreuss Water Power Gallery Tunnel, UPH-3, Venetia Mine, Vomp Eeast Tunnel, Whakapapa-Tawhitikuri Tunnel, Wheeler River Project, Yanzhou Coalfield

**Online-only Table 2:** Description of database fields for data records. The first table column (“#“) refers to data field sequence in a data record. It is the column number in spreadsheet version.

#	Label	Units	Description
1	Dataset #		dataset ID number
2	Category of research		one of five main categories
3	Site name		site name
4	Country		country name
5	Admin. region		administrative district, state, etc.
6	Locality name		place name (e.g. village, town, city)
7	Geologic region		geologic feature or region (e.g. sedimentary basin, mountain massif)
8	Geographic region		name of geographic feature or region
9	Latitude	degrees	geographic latitude in decimal degrees (degrees North are positive)
10	Longitude	degrees	geographic longitude in decimal degrees (degrees West are negative)
11	Elevation of ground on land	meters	land elevation above sea level, as reported
12	Elevation of sea floor	meters	sea floor elevation relative to sea level (negative values only)
13	Location uncertainty		note about location uncertainty
**Geologic summary**			
14	Geology - summary		brief text summary with references
15	Lithology of host rock		short text
16	Siliciclastic coarse	(1 = yes)	representative host rock type for fault zone tested
17	Mudrocks	(1 = yes)	representative host rock type for fault zone tested
18	Carbonates & evaporites	(1 = yes)	representative host rock type for fault zone tested
19	Volcaniclastic	(1 = yes)	representative host rock type for fault zone tested
20	Volcanic igneous	(1 = yes)	representative host rock type for fault zone tested
21	Plutonic	(1 = yes)	representative host rock type for fault zone tested
22	Metamorphic	(1 = yes)	representative host rock type for fault zone tested
23	Unlithified siliciclastic	(1 = yes)	representative host rock type for fault zone tested
24	Deformation bands only	(1 = yes)	this site has permeability of deformation bands only (not fault zone)
25	Structures - summary		brief text summary with references
26	Fault rocks - summary		brief text summary with references
**Fault types and sizes**			
27	Normal	(1 = yes)	predominantly dip-slip fault
28	Reverse	(1 = yes)	predominantly reverse fault (at present)
29	Normal & oblique	(1 = yes)	normal fault with significant lateral slip component
30	Thrust	(1 = yes)	thrust fault
31	Strike-slip	(1 = yes)	dominant strike-slip of fault
32	Fault along dyke or vein	(1 = yes)	any fault that follows fractured dyke or wide vein
33	High angle dip	(1 = yes)	for fault dip > 45 degrees or qualitative (e.g. steeply dipping fault)
34	Low angle dip	(1 = yes)	dip < 45 degrees or qualitative (e.g. shallow-dipping fault)
35	Active fault	(1 = yes)	as determined by active fault studies in this area
36	Dip	degrees	positive degrees from horizontal plane; stored as text if range of values
37	Throw	meters	dip-slip displacement only
38	Displacement	meters	total displacement of fault segment or fault zone
39	Length	meters	length of fault segment or fault zone
40	Fault core width	meters	widest measured at or near test site
41	Fault zone mapped width	meters	width of exposure on outcrops
42	Minimum fault zone total width	meters	inferred from partially covered outcrop, etc.
43	Maximum fault zone total width	meters	whole fault zone
**Permeability test methods summary**			
44	Permeability test methods		description of the main test methods (*in-situ* or on samples)
45	Core plugs or smaller samples	(1 = yes)	tested in laboratory permeameters (confined pressure)
46	Whole drill-core pieces	(1 = yes)	tested in laboratory permeameters (confined pressure)
47	Outcrop or drillcore spots using gas injection	(1 = yes)	probe permeameters
48	Infiltration or exfiltration at outcrop	(1 = yes)	water injected or removed from outcrop
49	Water inflows/outflows in drillholes	(1 = yes)	water circulation losses, feed zones, temperature anomalies
50	Spring water or gas flow from faults	(1 = yes)	thermal spring discharge, gas fluxes
51	Water inflow from fault zone to tunnel	(1 = yes)	mapped fault zones and observed water inflow rates
52	Single borehole tests	(1 = yes)	e.g. injection, slug/pulse tests
53	Tidal analysis	(1 = yes)	earth tides, ocean tides in drillholes in fault zone
54	Barometric fluctuations in unsaturated zone	(1 = yes)	modelling of air flow in fault zones
55	Cross-borehole tests in fractured zones	(1 = yes)	fracture networks tested between two drillholes
56	Pumping or injection tests	(1 = yes)	e.g. interference tests
57	Reservoir production/reinjection	(1 = yes)	production and re-injection data, transmissivity of whole site
58	Tracer tests and analysis	(1 = yes)	estimate of porosity, porosity-thickness, connectivity
**Other permeability estimation methods that are not hydraulic tests or permeameter tests**			
59	Other estimation methods		text summary
60	Microfracture or pore geometry	(1 = yes)	e.g. porosity-permeability relations
61	Empirical laws for fracture aperture and spacing	(1 = yes)	e.g. cubic law
62	Grout injection volume patterns	(1 = yes)	grout volume loss pattern across fault zone
63	Geophysical methods in drillholes	(1 = yes)	e.g. tube waves
64	Geochemical/mineralogical methods	(1 = yes)	e.g. paleo-permeability, paleo-fluid flux
65	Microseismicity migration over time	(1 = yes)	permeability estimated from hydraulic diffusivity
66	Thermal water upflow velocity	(1 = yes)	1D flow driven by temperature gradient and buoyancy
67	Numerical flow model calibration	(1 = yes)	fault zone and host rock permeability adjusted to calibrate model
**Permeability results review**			
68	Permeability results - summary		text summary, referenced to original authors
69	Initial review pass	(1 = yes)	result of this review
70	Review comments		notes about the initial review pass result
71	Reference list		selected reference list
72	Outcrop site	(1 = yes)	rock samples were tested or extracted from and tested in laboratory
73	Outcrop data used	(1 = yes)	outcrop permeability data are for a site that passed the initial review
74	Downhole *in-situ* data used	(1 = yes)	*in-situ* bulk permeability data are for a site that passed the initial review
**Depth information for** ***in-situ*** **interval or sample**			
75	Max depth	meters	maximum vertical depth below ground (1 at outcrops)
76	Min depth	meters	minimum vertical depth below ground for *in-situ* test intervals or zones
77	Average depth	meters	average vertical depth below ground for *in-situ* test intervals or zones
78	Depth to top of basement rock	meters	0 = bedrock outcrop, blank = unknown or not available during review
79	Max depth below top of basement	meters	maximum depth - top of basement depth
80	Min depth below top of basement	meters	minimum depth - top of basement depth
81	Average depth below top of basement	meters	average depth - top of basement depth
82	Max elevation	meters	*in-situ* fault zone (top of interval)
83	Min elevation	meters	*in-situ* fault zone (bottom of interval)
84	Average elevation	meters	*in-situ* fault zone (mid point of interval)
85	Max confining pressure on samples	MPa	maximum confining pressure in laboratory
86	Min confining pressure on samples	MPa	minimum confining pressure in laboratory
87	Confining pressure for effective depth	MPa	confining pressure in laboratory that is used to estimate effective depth
88	Effective depth for samples	meters	effective depth for permeability value (not sample depth)
89	Confining P» P at sample depth	(1 = yes)	if the effective depth at confining pressure» sample depth
**Fault core zone (FC) permeability (k)**			
90	FC matrix k	log(m^2^)	matrix permeability of fault rock in the fault core zone
91	FC matrix k low estimate	log(m^2^)	matrix permeability of fault rock in the fault core zone
92	FC bulk k	log(m^2^)	bulk permeability (*in-situ* or estimated) in the fault core zone
93	FC bulk k low estimate	log(m^2^)	bulk permeability (*in-situ* or estimated) in the fault core zone
**Fault damage zone (DZ) matrix permeability (k)**			
94	DZ matrix k	log(m^2^)	matrix permeability in the fault damage zone
95	DZ matrix k low estimate	log(m^2^)	matrix permeability in the fault damage zone
**Fault zone (FZ) bulk permeability (damage zone or combined damage zone + fault core)**			
96	* FZ bulk k	log(m^2^)	bulk permeability from *in-situ* tests or estimates for the fault zone (see column 98 and 99 categories whether the permeability is specific to damage zone only)
97	* FZ bulk k low estimate	log(m^2^)	bulk permeability from *in-situ* tests or estimates for the fault zone (see column 98 and 99 categories whether the permeability is specific to damage zone only)
98	FZ test only in damage zone	(1 = yes)	FZ bulk k values in column 96 and 97 represents fault damage zone only
99	FZ test in damage zone may include fault core	(1 = yes)	FZ bulk k values in column 96 and 97 represents fault damage zone and may include parts of fault core(s)
**Host rock (naturally fractured protolith) permeability**			
100	Host rock matrix k	log(m^2^)	samples from host rock (protolith)
101	Host rock matrix k low estimate	log(m^2^)	samples from host rock (protolith)
102	Host rock bulk k	log(m^2^)	*in-situ* tests or estimates for naturally fractured host rocks
103	Host rock bulk k low estimate	log(m^2^)	*in-situ* tests or estimates for naturally fractured host rocks
**Ratios of log permeability values (FZ = fault zone, FC = fault core, FD - fault damage zone, HR = host rock or protolith)**			
104	log(FZ/HR), for bulk k		log(m^2^/m^2^)… (fault zone/host rock), bulk k
105	log(FZ/HR), for bulk k low estimates		(fault zone/host rock), bulk k
106	log(FC/HR), for matrix k		(fault core/host rock), matrix k
107	log(FC/HR, matrix k low estimates		(fault core/host rock), matrix k
108	log(FC/HR), for bulk k		(fault core/host rock), bulk k
109	log(FC/HR), for bulk k low estimates		(fault core/host rock), bulk k
**log values of permeability**			
110	log FC matrix k	log(m^2^)	matrix permeability of samples from fault core zone
111	log FC matrix k low estimate	log(m^2^)	matrix permeability of samples from fault core zone (low estimate)
112	log FC bulk k	log(m^2^)	*in-situ* tests or estimates for fault core zone
113	log FC bulk k low estimate	log(m^2^)	*in-situ* tests or estimates for fault core zone (low estimate)
114	log DZ matrix k	log(m^2^)	damage zone matrix permeability (samples or outcrop)
115	log DZ matrix k low estimate	log(m^2^)	samples from fault damage zone, but of cohesive rock matrix only
116	log FZ bulk k	log(m^2^)	*in-situ* tests representative estimates for fault zone
117	log FZ bulk k low estimate	log(m^2^)	*in-situ* tests representative estimates for fault zone (low estimate)
118	log HR matrix k	log(m^2^)	samples from host rock (protolith)
119	log HR matrix k low estimate	log(m^2^)	samples from host rock (protolith)
120	log HR bulk k	log(m^2^)	*in-situ* tests or estimates for naturally fractured host rocks
121	log HR bulk k low estimate	log(m^2^)	*in-situ* tests or estimates for naturally fractured host rocks
